# Spontaneous assembly of a hybrid crystal-liquid phase in inverse patchy colloid systems†
†Electronic supplementary information (ESI) available. See DOI: 10.1039/C6NR07987C



**DOI:** 10.1039/c6nr07987c

**Published:** 2016-12-20

**Authors:** Silvano Ferrari, Emanuela Bianchi, Gerhard Kahl

**Affiliations:** a Institut für Theoretische Physik , TU Wien , Wiedner Hauptstraße 8-10 , A-1040 Wien , Austria . Email: emanuela.bianchi@tuwien.ac.at; b Institut für Theoretische Physik and Center for Computational Materials Science (CMS) , TU Wien , Wiedner Hauptstraße 8-10 , A-1040 Wien , Austria . Email: gerhard.kahl@tuwien.ac.at

## Abstract

Materials with well-defined architectures are heavily sought after in view of their diverse technological applications. Among the desired target architectures, lamellar phases stand out for their exceptional mechanical and optical features. Here we show that charged colloids, decorated on their poles with two oppositely charged regions possess the unusual ability to spontaneously assemble in different morphologies of (semi-)ordered, layered particle arrangements which maintain their structural stability over a surprisingly large temperature range. This remarkable capacity is related to a characteristic bonding mechanism: stable *intra-layer* bonds guarantee the formation of planar aggregates, while strong *inter-layer* bonds favor the stacking of the emerging planar assemblies. These two types of bonds together are responsible for the self-healing processes occurring during the spontaneous assembly. The resulting phases are characterized by parallel, densely packed, particle layers connected by a relatively small number of intra-layer particles. We investigate the properties of the (semi-)ordered phases in terms of static and dynamic correlation functions, focusing in particular on a novel hybrid crystal-liquid phase that prevails at intermediate temperatures where the inter-layer particles form a mobile, fluid phase.

## Abstract 2

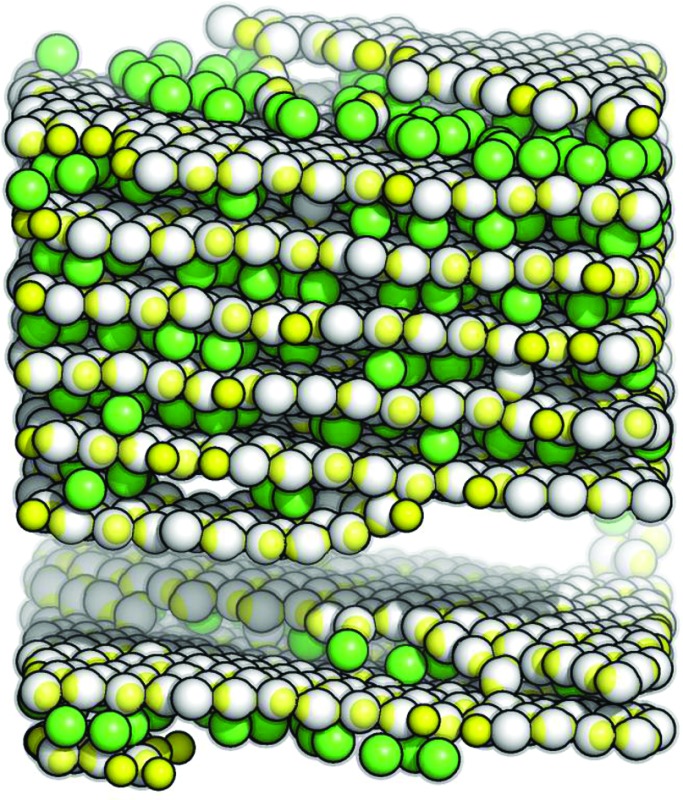
Heterogeneously charged colloids with a simple surface charge distribution are able to spontaneously form a phase of parallel, close-packed monolayers spaced by mobile inter-layer particles that is stable over a wide temperature range.

## 1. Introduction

Most of the present strategies for the fabrication of materials with desired physical properties and/or specific responses to external stimuli rely on the self-assembly of mesoscopic target architectures from suitably designed building blocks.^1,2^ Among the desired target structures, layered phases rank at a very prominent position due to their outstanding features.^3^ Lamellar systems have indeed exceptional mechanical and optical properties that make them relevant for a wealth of technological applications such as data storage devices, high-surface-area catalysts, lubricants and photoconductors.^3–8^ A very interesting layered phase with applications in gene therapy is a lamellar structure with alternating lipid bilayers and intercalated DNA monolayers.^9,48^ Layered structures are typically self-assembled from surfactants, lipids or block copolymers where the double nature of these molecules plays a key role in their ordering behavior;^10^ layered phases are also observed in other naturally occurring systems, such as S-layer proteins,^11^ rod-shaped viruses^12^ and clays,^13^ as well as artificial nacre-like systems,^14^ where lamellar phases originate mainly from dipolar interactions, anisotropic shapes or site-specific binding.

In the realm of colloidal particles, the template-free assembly of two-dimensional sheets is a very challenging task which is nevertheless highly rewarding in view of the many technological applications of such phases. Anisotropy – be it in the shape and/or in the surface patterns of the particles – has turned out to be a very versatile and powerful instrument to foster and speed-up self-assembly processes in both three and two dimensions.^15,16^ Strategies based on anisotropy-driven self-assembly have gained an additional boost from a rapid progress in the manufacturing and synthesis techniques of the individual building blocks, from anisometric colloidal particles^17–20^ to spherical colloids with directional effective interactions such as dipolar or multipolar particles,^16^ DNA-decorated colloids^21,22^ and systems with heterogeneously patterned surfaces, often referred to as patchy colloids.^23,24^ Patchy particles are characterized by well-defined bonding sites on an otherwise repulsive surface. Material production methods based on anisotropy-driven self-assembly have been used to fabricate colloidal mono- and double-layers with alloy nanoparticles (*e.g.*, CdTe,^25^ PbS^26^ and PbSe^27^), Janus particles,^28,29^ trivalent patchy particles^30^ and particles with multiple patches.^31^

Recently, the formation of colloidal two-dimensional structures has also been observed in systems of particles with heterogeneously charged surfaces, referred to as inverse patchy colloids^32^ (IPCs). The adjective “inverse” characterizes the bonding behavior of IPCs as opposed to conventional patches: depending on their relative orientation two interacting particles experience a repulsion if regions of like charge face each other and an attraction otherwise.^33^ Originally introduced as a theoretical model to describe particles characterized by a non-homogeneous surface charge, IPCs have been meanwhile synthesized in the lab.^34^ Both experiments and simulations have provided evidence that IPCs carrying two identical polar patches and an oppositely charged equatorial belt are able to form planar assemblies with well-defined translational and orientational order when they are close to a weakly homogeneously charged substrate.^34–36^ This tendency to form layered structures has also been observed in the bulk: crystal structures composed of parallel monolayers were found to be stable for a wide variety of characteristic system parameters and over a large range of external physical conditions.^37,38^ In addition, the control over the system behavior could be achieved *via* conveniently accessible experimental parameters such as the pH of the solution and/or the presence of salt in the system.^38^

In contrast to many of the layer-forming systems, the lamellar solid formed by IPC systems so far^37,38^ combines three important features: (i) it is an equilibrium phase, (ii) it is assembled in pure one-component systems and (iii) it is characterized by a non-close-packed structure, *i.e.* the colloidal monolayers are not stacked in direct contact on the top of each other. The latter feature is a consequence of the characteristic bonding mechanism between IPCs that is also responsible for the internal stability of the layers: bonds between IPCs form *via* an equator–patch contact when the particle axes lie within the planes, exposing thereby their equatorial belts to the neighboring layers. Within this structure,^37,38^ the equilibrium distance between adjacent layers depends on the thermodynamic conditions and can thus be possibly quite large in some cases. While the self-assembly of isolated planar sheets has been observed in many bulk IPC systems, the spontaneous formation of an extended stacking of non-close packed monolayers is hard to be observed in unbiased many body simulations due to the intrinsic nature of the described lamellar phase.

**Fig. 1:**
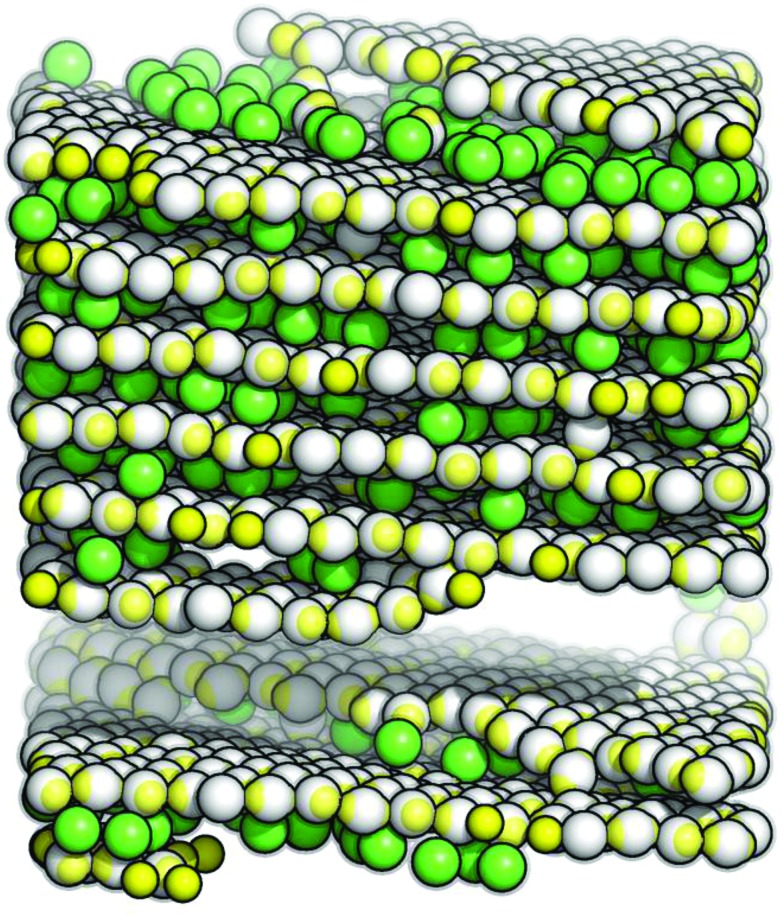
Snapshot of a self-assembled hybrid crystal-liquid phase: the layered phase is composed of parallel hexagonal layers (grey particles) and up-right, mobile inter-layer particles (green particles). State point: ρ* = 0.70 and T* ≈ 0.060.

Here we present a new type of layered architecture that is observed to spontaneously assemble: in contrast to the previously observed lamellar phase,^37,38^ the stability of the present structure is reinforced by inter-layer particles that show a well-defined stoichiometric ratio with respect to the particles that populate the layers. While the intra-layer particles guarantee the formation of extremely stable layers, the inter-layer particles are able to fix the equilibrium distance and the mutual orientation of adjacent monolayers. The self-assembly of such an architecture is guaranteed by a characteristic bonding mechanism: the present IPCs are able to form at the same time not only (i) strong *intra-layer* bonds (realized by particles oriented in the planes) but also (ii) strong *inter-layer* bonds (realized by particles placed between two adjacent layers and oriented perpendicularly to the planes). The combination of these two bonding mechanisms guarantees the high stability of different types of emerging layered structures over a remarkably large temperature range: while at low temperatures a fully ordered structure with a complex internal architecture is found to be stable, we observe at intermediate temperatures a novel hybrid crystal-liquid phase (see Fig. 1), where the inter-layer particles form a disordered, mobile phase that is confined between densely packed parallel layers, the latter ones being characterized by a strong intra-layer cohesion. Only at relatively high temperatures these bonds also break up and the system eventually melts. Thus the system undergoes *via* a steady temperature increase a very unique two-stage melting process.

## II. Model

A model for IPCs was first put forward in ref. 32: it views such a (mesoscopic) particle as a hard sphere of radius *σ*_c_ and charge *Z*_c_, decorated with two patches, each of charge *Z*_p_ and each located at a distance *e* from the colloid center, in opposite directions along the particle diameter. This geometry defines the two polar regions (occupied by the patches), while the remaining, bare colloidal surface is referred to as the equatorial region. The electrostatic screening of the surrounding (microscopic) solvent determines the interaction range *δ* of the IPC; *δ* and *e* together fix both the radius of the patch interaction spheres and the patch angular extent *via* eqn (10) and (11) of ref. 32. The energy strengths of the different interaction spheres in eqn (13) of ref. 32 are fixed *via* a mapping described in ref. 32 and used in ref. 39.

The diameter of the colloid is taken as the unit of length (2*σ*_c_ = 1); further, *δ*/2*σ*_c_ = 0.2 and *e*/2*σ*_c_ = 0.22, leading to a patch angle of 45.6°. The charges *Z*_c_ and *Z*_p_ are chosen such that a particle is overall neutral; *i.e. Z*_c_ + 2*Z*_p_ = 0. All energies are expressed in units of the minimum value of the equatorial–polar attraction, *ε*_m_, and inverse temperatures (1/*k*_B_ *T*) are expressed in this unit. Choosing the mass unit *m*_0_ such that each IPC has a mass of 3*m*_0_, the natural time unit is fixed *via* 
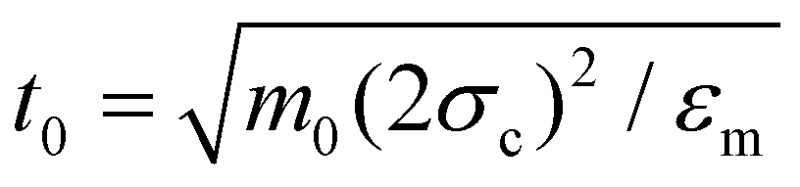
.

All observables are given in reduced units, defined as T* = *k*_B_ *T*/*ε*_m_, ρ* = *ρ*(2*σ*_c_)^3^ and t* = *t*/*t*_0_.

## III. Methods

Most of the results presented in this contribution are obtained in Molecular Dynamics (MD) simulations in the microcanonical (NVE) ensemble, using the velocity Verlet integration algorithm.^40^ In our simulation scheme the IPCs are modeled as stiff, linear molecules, composed of three massive interaction centers. Since the conventional RATTLE procedure to simulate molecules with geometric constraints^41^ is singular for linear particles,^42^ we calculate the dynamics of the patch-center-patch geometry of our particles by reducing the number of degrees-of-freedom *via* a scheme presented in ref. 42. All conceptual details of our implementation are presented in ref. 39. Systems of 1372 to 2000 particles (focusing on self-assembly) and of 1302 particles (for the ordered phases) have been considered. Time increments have been used that range between 2.5 × 10^–3^ *t*_0_ (at high temperatures) and 10^–3^ *t*_0_ (at low temperatures). Equilibration at low temperatures is achieved by starting from an equilibrated system at T* = 1.0 and then slightly lowering the temperature: the ratio between the new temperature and the old one is initially 0.5 and it smoothly increases on approaching the desired temperature. The locations of the gas-liquid critical point and the related coexistence line were estimated *via* grand-canonical Monte Carlo (GCMC) simulations using the histogram reweighting technique.^43^ Systems in a cubic box with side *L* = 22*σ*_c_ were considered over ten independent MC runs – each one extending over more than 10^6^ MC steps; more details have been presented in ref. 39. Direct coexistence MC simulations were used to study isolated state points: the layered structure and the fluid were put at contact in a rectangular box of size *L*_*x*_ = 10.4, *L*_*y*_ = 12.0 and *L*_*z*_ = 24.0 at several temperatures over 10^7^ MC steps. Several densities for the layered structure were considered (upon changing the number of inter-layer IPCs) and it was observed that at densities different from ρ* = 0.75, additional inter-layer particles in the crystal migrated to the fluid as well as lacking particles in the inter-layer space were captured from the fluid, thus confirming the fixed ratio between inter-layer and layer IPCs in the perfect crystal.

The dynamics was studied in terms of the mean squared displacement (MSD):

1 Δ*r*^2^(*t*) = 〈[*r*(*t*) – *r*(0)]^2^〉

and of the orientational autocorrelation function (OACF), defined as:

2 *C*_*n*_(*t*) = 〈*n*(*t*)·*n*(0)〉.

The consistency between MC simulations and MD simulations was checked by comparing energies, pair distribution functions and static structure factors at several equilibrium state points. No significant differences between the results obtained *via* the different algorithms were observed.

## IV. Results

### A. The layered structure

**Fig. 2:**
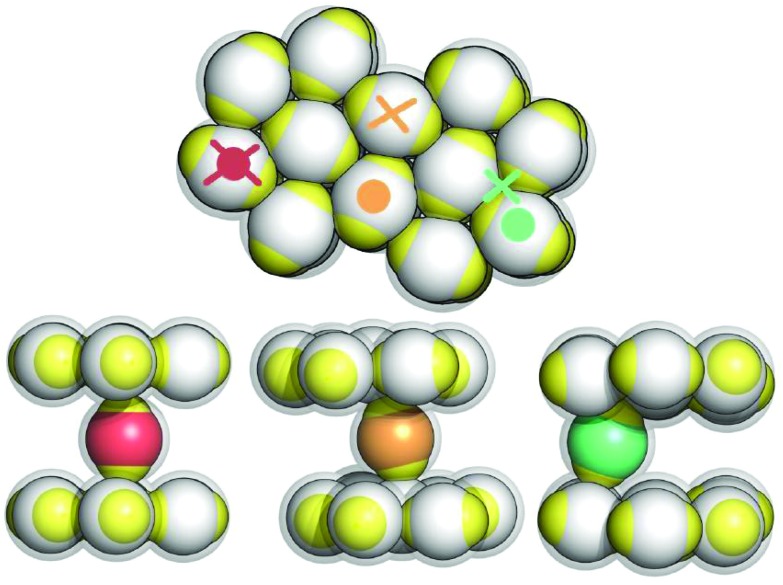
Schematic representation of the layered structure: the colloid centers of the layer particles are shown in gray, while the colloid centers of the inter-layer particles are specified in different colors according to their position/orientation (see text); throughout, the patches are colored in yellow; the light gray halo around the particles represents their interaction sphere. Top panel: view of the hexagonal layers; symbols represent the *x*–*y* position of the patches belonging to the selected inter-layer particles shown in the side views below: a full circle for the lower patch, a cross for the upper patch. Bottom panels: side views of three independent characteristic configurations, obtained from simulations at ρ* = 0.75 and T* = 0.015 (left panel) or T* = 0.030 (middle and right panels). The red color characterizes the ground state structure, while the orange and green colors specify configurations as they occur in two of the most common types of defects (see text).

We start our discussion of the results by describing the *fully ordered* layered phase. A representative portion of this structure is depicted in Fig. 2. The layered architecture is characterized by two types of particles: IPCs that belong to the layers are referred to as *layer particles*, while IPCs that connect two adjacent layers are called *inter-layer particles*. The former ones assemble into a close-packed, triangular lattice with a well-defined orientational pattern, where the symmetry axes of all the particles lie in the plane and only two particle orientations along parallel lanes of particles are observed (top panel of Fig. 2): particles pertaining to either of the lanes are oriented parallel to each other, while the orientational axes of the particles in different lanes enclose an angle of ≃86.4°; this particular geometrical arrangement guarantees a strong intra-layer bonding mechanism *via* an optimal equator–patch contact. We observe that even though a layer particle forms six bonds with its neighbors only four bonds occur in the optimal equator–patch arrangement, while the remaining two occur with a parallel alignment of the particles; thus the binding energy per particle does not amount to –3 but rather to ≃–2.35. Bonding between different layers is ensured by IPCs oriented orthogonally to the layers: the stoichiometry between the layer and inter-layer particles is given by a fixed ratio equal to 2/7 (see the ESI†); the inter-layer particles are located in direct contact with the layer particles of the confining lower and upper layers, so that the crystal density amounts to ρ* ≃ 0.75. Ideally, an inter-layer particle establishes (*via* two equator–patch contacts) a bond between two adjacent planes that are perfectly flat and aligned, resulting in a binding energy equal to –1.

At the lowest temperature investigated, the system is able to stabilize the described structure essentially without any defects, so that we can refer to this arrangement as the ground state configuration of the system. A representative section of this fully ordered crystal is depicted in the bottom/left panel of Fig. 2 (in red). At higher temperatures, however, some defects can occur: while vacancies within the layers are rare, we typically observe that the layers can be either not exactly aligned or not perfectly parallel; then inter-layer IPCs have to tilt from the ideal, orthogonal orientation in order to maximize the bonding strength: when the layers are shifted with respect to each other, inter-layer particles bend to maximize the equator–patch overlap volume, so that the bonds are still realized *via* a direct contact between the equator of a layer particle and the patch of an inter-layer particle, as shown in the bottom/middle panel of Fig. 2 (in orange); on the other hand, when the layers are not perfectly straight (*i.e.* the distance between the layers is reduced), steric requirements force the inter-layer particles to have only one equator–patch bond, while the other patch is located at the center of a triangle formed by the closely packed layer particles, as depicted in the bottom/right panel of Fig. 2 (in green).

### B. Phase diagram

**Fig. 3:**
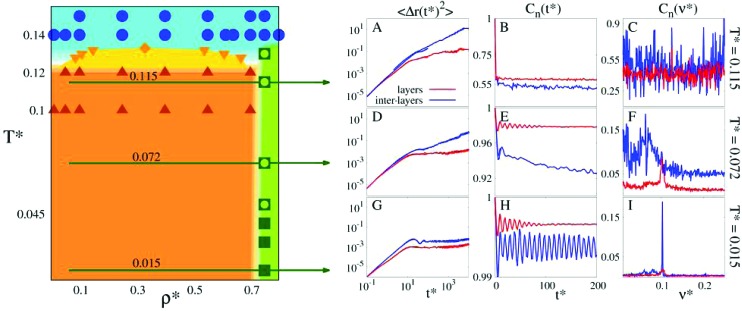
Main panel (left): estimate for the temperature T* *vs.* density ρ* phase diagram of the system at hand. The orange area corresponds to the coexistence region between the vapor and the layered phases, while the yellow area indicates the liquid–vapor coexistence region; areas where the fluid phase is stable are depicted in light blue; green specifies the region of stability of the layered phases. Symbols mark state points that have been explicitly investigated in simulations: blue circles for the fluid phase; brown, upright triangles for state points where the vapor and the layered phases coexist; orange, downright triangles for the liquid–vapor coexistence line; orange diamond for the liquid–vapor critical point; empty green squares for the hybrid crystal-liquid phase; full green squares for the fully ordered, layered structure. Small panels (right): MSD 〈Δ*r*(*t*)^2^〉 (panels A, D, G), OACF *C*_*n*_(*t*) (panels B, E, H) and its Fourier transform *C*_*n*_(*ν*) (panels C, F, I), as labeled. The respective functions of the layer and the inter-layer particles are shown in different colors, as labeled. Panels A, B and C: T* = 0.115; panels D, E and F: T* = 0.072; panels G, H and I: T* = 0.015 (as emphasized by the green horizontal arrows).

Due to the high computational cost, we refrain from an accurate determination of the phase diagram, but we rather provide an estimate for those regions in the (T*,ρ*)-plane where the fluid and the layered phases are found to be stable or where they coexist. The semi-quantitative phase diagram, reported on the left panel of Fig. 3, is dominated by a wide region (marked in orange) where the gas phase (occurring only at very small densities, *i.e.*, ρ* ≪ 0.01) coexists with two distinct layered solid phases (both at ρ* ≃ 0.75). Furthermore, the phase diagram shows a rather flat vapor–liquid coexistence curve (marked in yellow): the critical point is located at 
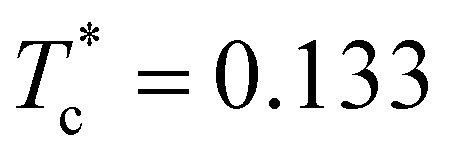
 and 
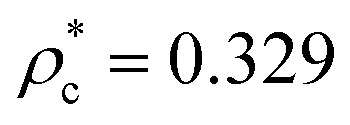
. The fully ordered layered architecture depicted in Fig. 2 (and discussed above) spontaneously forms at very low temperatures (T* ⪅ 0.045), while at T* ≃ 0.045 the inter-layer particles become mobile, leading to a hybrid crystal-liquid structure; this *semi-ordered* phase is characterized by a partial transport of the inter-layer particles between the parallel planes. Eventually, at T* ≃ 0.135 the strong intra-layer bonds also break up and the system melts completely.

### C. Phenomenological analysis of the ordered structures

The aforementioned two layered phases that our system is able to form in the respective temperature ranges may be better appreciated by resuming our discussion with the observations made at the lowest temperatures investigated: here the strategy of the system to form highly-stable layered structures becomes most apparent. For a more quantitative analysis we focus now on the mean squared displacement (MSD) and on the orientational autocorrelation functions (OACFs) – *i.e.* the autocorrelation functions of the unit vector *n* connecting the particle center with one of its polar patches – as functions of both time and frequency; each of these quantities is displayed separately for the layer and for the inter-layer particles (see Methods).

At the lowest temperature investigated, T* = 0.015, both types of particles are positionally frozen and thus do not diffuse, as reflected by the time-dependence of the MSDs depicted in panel G of Fig. 3: beyond the ballistic regime, the MSDs of both types of particles soon level off, attaining in the long-time limit two slightly different values. Since both values correspond to distances that are much smaller than the average spacing between the particles, we can conclude that at such a low temperature essentially no particle diffusion takes place. The OACFs of both types of particles (displayed in panel H of Fig. 3) are characterized by an oscillatory, non-decaying behavior as functions of time indicating that both layer and inter-layer particles oscillate around their equilibrium positions.^44^ The respective Fourier transforms of the OACFs (shown in panel I of Fig. 3) clearly display a sharp peak both at the same frequency.

The depicted scenario is essentially unchanged for temperatures up to T* ≃ 0.045, indicating that the system preserves its complex layered structure. At this temperature the system undergoes the first structural transition of the above mentioned two-stage melting process. For temperatures above this threshold value and below T* ≃ 0.135, the inter-layer particles gain more and more mobility and eventually start to move freely between the layers; in contrast the thermal energy is not yet sufficiently large to break up the strong bonds formed within the layers. This scenario guarantees that the layered structure of the system is rigorously preserved: the emerging phase can be viewed as a unique hybrid crystal-liquid structure, where the inter-layer particles form a disordered, mobile phase, that is confined between the persisting, essentially rigid and highly stable structure of the parallel layers.

A closer inspection of the dynamic correlation functions at T* = 0.072 (displayed in panels D to F of Fig. 3) consistently confirms this scenario: the MSD of the inter-layer particles shows a linear increase as a function of time, providing evidence that diffusion does occur for these particles; further, by separating the MSD of the inter-layer IPCs in directions parallel and perpendicular to the layers (see the ESI†), we observe that particle diffusion takes place exclusively parallel to the planes (which obviously form impenetrable walls for the mobile particles). On the other hand, the MSD of the layer particles is still rather flat and attains – within the observation period – values that hardly exceed 10^–2^; this lack of diffusion of the layer particles (which confirms the rigidity of the layered structure) persists over a surprisingly large range in temperature, *i.e.*, up to T* ≃ 0.135. The OACF of the layer particles is still an oscillating function that does not decay in time; from its spectrum we learn that the particles oscillate around their equilibrium orientations with the same frequency as the one observed for T* ≃ 0.015. In contrast, the OACF of the inter-layer particles shows a significant decay in time. As we steadily increase the temperature, the OACF preserves its oscillatory character. Together with the translational diffusion, these oscillations lead to a characteristic “dancing” movement of the particles. The dynamics of the inter-layer particles is further investigated *via* the energetic analysis reported in the next section. Eventually, for higher temperatures (but still below T* ≃ 0.135), the spectrum of the inter-layer particle OACF has lost any significant traces of characteristic oscillations, indicating – in combination with the increasing MSD – a purely diffusive behavior of these particles. Additional information on other observables that support the depicted scenario is shown in the ESI.†

Eventually, for T* ⪆ 0.135 the thermal energy becomes sufficiently high to break up also the tight intra-layer bonds and the system melts completely (panels A to C of Fig. 3), realizing thus the second stage of the two-stage melting process.

With the above discussion in mind, it is worth emphasizing once again the surprisingly high persistence of the hybrid crystal-liquid phase, which extends over a remarkably large temperature range (*i.e.*, 0.045 ⪅ *T* ⪅ 0.135); this characteristic feature is uniquely due to the ability of IPCs to form selective and highly directional bonds with neighboring particles. Thus within a relatively broad temperature range, the spontaneous formation of the underlying, highly stable (semi-)ordered architecture offers the possibility of particle transport through a well-defined geometry, formed by stacked, parallel and essentially rigid layers. While in our system both mobile and confining particles are realized by the same species of colloids, one might consider in future, other mobile particles, characterized by more exotic and/or versatile features that are, for instance, able to perform carrier tasks.

### D. Energetic considerations

The equilibrium oscillations of the inter-layer particles in the regime where the fully ordered crystal is observed (*i.e.* for T* ⪅ 0.045) – displayed in panels H and I of Fig. 3 – call for a deeper analysis. The dynamics of the inter-layer particles within the crystal indeed turns out to be a complex, “dance”-type motion that is induced by the emergence of small defects in the ground state structure as the temperature is increased. The ground state architecture is characterized by perfectly aligned, parallel layers which are separated by two particle diameters; thus an inter-layer particle connecting the equatorial belts of two layer particles is oriented perpendicular to the planes and is placed in direct contact with the layers. As the temperature rises, the mobility of the particles increases, leading to (i) relative horizontal shifts of adjacent layers (typically up to 30–50% of the particle diameter) and/or (ii) small local deviations from the perfect inter-layer distance. As a consequence, the inter-layer IPCs are forced to (i) tilt with respect to the ideal, perpendicular orientation and/or (ii) move from their stable equilibrium positions to slightly less stable ones.

**Fig. 4:**
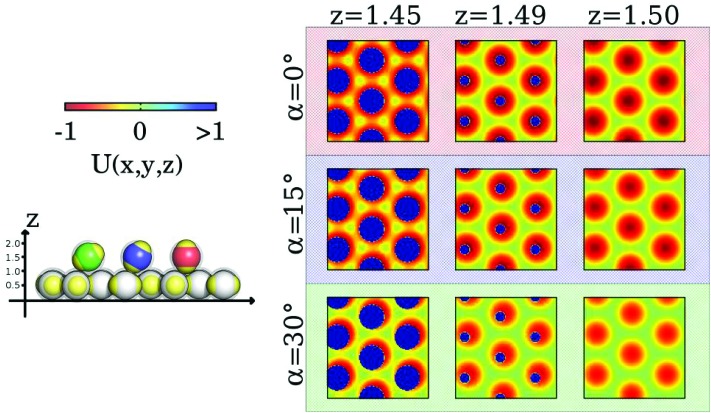
Left: Color scale of the energy contour plots (top) and a schematic representation that visualizes the orientations of the inter-layer particles by coloring them according to *α* (bottom). Right: Contour plots of the potential energy landscape, *U*(*x*, *y*, *z*), probed by a single inter-layer IPC with different orientations and different distances from an adjacent plane. We report a grid of nine contour plots where the rows refer to three different *α* = 0°, 15° and 30° (as labeled) and the columns refer to three different *z* = 1.45, 1.49, 1.50 in units of the particle diameter (as labeled). The blue areas correspond to forbidden regions due to the steric repulsion between IPCs, *i.e. U*(*x*, *y*, *z*) ≫ 1.

The inter-layer particle dynamics can be better understood by investigating the energy landscape experienced by a tagged inter-layer particle in the presence of a nearby layer as its orientation and position vary. The ensuing variation of this potential energy is shown in the contour plots of Fig. 4: here we vary the horizontal position of a probe inter-layer IPC (in terms of its *x*- and *y*-coordinates) with a given orientation *α* (with respect to the direction perpendicular to the plane) and a fixed vertical distance *z* from the nearby layer: three characteristic values for *α* (*i.e.*, 0°, 15°, and 30°) and three representative values for *z* (*i.e.*, 1.45, 1.49, and 1.50) are considered.

The color-coded energy landscape is shown in the panels of Fig. 4. From these contour-plots it can be inferred that the energetically most favorable particle arrangement is achieved when the tagged particle aligns perfectly orthogonal to the plane and when its center is located at *z* = 1.5, placing its patches on top of the equators of the layer particles (top-right panel): the resulting equator–patch bond corresponds to the lowest possible bond strength, *i.e.* –1 (dark red color code). This (essentially ideal) configuration is observed at rather low temperatures, when almost no defects are present. As *z* is decreased (moving along a row of panels from the right to the left) strongly repulsive circular regions (dark blue circles) emerge due to the steric effects imposed by the impenetrable cores of the particles. At any *α*-value, these repulsive regions grow in extent as *z* is decreased. The repulsive areas are surrounded by attractive coronas (red color): these energetically favorable areas are separated by regions with vanishing potential energy, reflected in the plots by greenish areas with a six-fold symmetry. As we now increase *α* (*i.e.*, moving along a column of panels from the top to the bottom) the extent of the annulus-shaped, attractive regions is reduced.

Since the contribution of a perfectly aligned top layer to the contour plots would add consistently to the contribution of the lower layer, from the above discussion we can infer that a center-to-center distance between two layer particles that is less than two particle diameters forces the inter-layer particles to slightly tilt out of their ideal orientation so that they can take advantage of the attractive, annulus-shaped regions, minimizing their energy by forming bonds with the layer particles as described in the bottom/right panel of Fig. 2. However, at low temperatures particle transport is still suppressed by the relative energy barriers separating the annulus-shaped regions. Thus particles perform a “dance”-type motion in these coronas. However, when provided by a sufficient amount of thermal energy (*i.e.* on increasing the temperature), particles are able to move/hop from one annulus to an adjacent one: under these conditions particle transport can be realized. It is also worth noting that, since tilting decreases the depth of the potential well of the annulus, it also reduces the height of the relative potential barriers between different annuli; this confirms that, as we observed in the simulations, tilting of the IPCs plays an important role in the diffusion process.

While for the case of two perfectly aligned layers it is sufficient to consider the energy landscape of the bottom layer, in the case that two adjacent planes are not perfectly aligned the energy landscape that a tagged particle experiences results from the superposition of the slightly misaligned contour plots of the two layers. In the resulting landscape one would observe the emergence of non-symmetric, half-moon shaped, attractive areas located around the repulsive cores. Nonetheless, these modifications in the energy do not change the oscillating dynamics of the particles significantly. In the case that two adjacent layers are not perfectly aligned, the patches of the inter-layer particles can still form stabilizing bonds with the equators of the respective layer particles by tilting their symmetry axes as shown in Fig. 2, bottom/middle panel.

### E. Outlook & summary

We have provided evidence that colloidal particles carrying a specific surface decoration – realized by two charged, polar patches (with a moderate opening angle) and an oppositely charged equatorial belt – are able to spontaneously form ordered layered structures which maintain their thermal stability over a surprisingly large temperature range. These observations – based on extensive MD and MC simulations – are more astonishing as this self-assembly scenario can be realized by involving only one single species of particles, while many layered structures usually require at least two particle species.

The key feature that guarantees the formation of the lamellar phases is the capacity of our particles to form selective and highly orientational bonds: these strong ties are realized by a close contact between the polar regions and the oppositely charged equatorial belts of neighboring particles. As these bonds occur for particles within the layers as well as for the connecting inter-layer particles, their combined occurrence guarantees the remarkable stability of the emerging layered structure, in particular at low temperatures: here particles oscillate at a specific frequency around their equilibrium orientation, but are essentially frozen in their positions. As we now increase the temperature, the system partially melts: the inter-layer particles become mobile and start to propagate along the complex energy landscape, formed by the still stable, hexagonally close-packed, adjacent layers. This unique hybrid crystal-liquid phase remains stable on further increasing the temperature and thus an essentially unhindered transport of the inter-layer particles is observed. Eventually, when the temperature is sufficiently high, the system undergoes the second step in the two-stage melting process and melts completely.

The fundamental role of the bonding mechanism described above, which is of particular relevance at low temperatures, can be appreciated during the simulation runs (see the ESI† and the related movie): by starting from a completely random particle configuration, we first observe the formation of hexagonally closed-packed particle sheets which are not aligned parallel to each other but are rather randomly distributed in space; then, the yet free particles start to play their role as connectors: once they are adsorbed on one of the layers they serve as anchoring points for the next parallel layer. By this mechanism, the ordered lamellar structure is built up layer by layer. This process turns out to be so efficient that even misaligned planes dissolve in favor of existing stacked layers. On the other hand, destroying intentionally such a structure provokes the same mechanism: the original structure is quickly restored, proving thereby the strong self-healing capacities of these particles.

We note that inverse patchy colloids are a class of multipolar particles; other multipolar units are currently investigated by other groups with different underlying models; however their self-assembly is often guided by the presence of external fields (see, *e.g.*, ref. 45 and 46 and references therein).

Experimental realizations of IPCs suggest that our idealized model represents the most simple picture of the particles as they are synthesized in the lab, where asymmetry in the patch extent and/or deviations from the overall neutrality of the particles is observed. Preliminary investigations with variations of the original model that mimic the experimental features reveal that the self-assembly of the observed lamellar phase is quite robust with respect to variations of the model parameters.^47^ In particular, as long as the changes in the patch extent are not dramatic, the layered structure maintains its stability even when some asymmetry between the patches (in size and/or charge) is introduced. Nonetheless, a patch opening angle around 45° seems the best choice to guarantee a remarkably high stability of the layered structure. Additionally, a slight over-charge of the particles (with the net charge being of the same sign as the equatorial charge) leads to an even stronger tendency of layer formation and to more stable layered structures.

To summarize, in contrast to conventional patchy colloids, inverse patchy units are able to spontaneously assemble into a stable, non-close-packed, lamellar phase, where the distance between two adjacent layers is determined by the synthesis process of the colloidal particles (since it is fixed by the size of the particles themselves). Additionally the particular two-stage melting process identified for our system is unique and unprecedented for colloidal particles with heterogeneously patterned surfaces and offers many possibilities for technological applications: in case the inter-layer particles are mobile, the lamellar phase as a whole is a rather anisotropic material being solid in the direction normal to the layers while essentially liquid in the two other directions, as the planes are free to slide over each other in response to shear.

We note that considering long range electrostatic interactions – and the ensuing correlations between the positions of the inter-layer particles belonging to different layers – might lead to even more exotic scenarios.^9^
