# Supplementary material for: Spontaneous assembly of a hybrid crystal-liquid phase in inverse patchy colloid systems
Source: Nanoscale. 2016 Dec 20;9(5):1956–63. doi: 10.1039/c6nr07987c (PMC5315017; doi:10.1039/c6nr07987c)
Supplement: Supplementary file 1 [file NR-009-C6NR07987C-s001.pdf]

Electronic Supplementary Material (ESI) for Nanoscale.
This journal is © The Royal Society of Chemistry 2016

Spontaneous assembly of a hybrid crystal-liquid phase in inverse patchy colloid systems – Supplementary Material

Silvano Ferrari and Emanuela Bianchi*

Institut für Theoretische Physik, TU Wien,

Wiedner Hauptstraße 8-10, A-1040 Wien, Austria

Gerhard Kahl†

Institut für Theoretische Physik and Center for Computational Materials Science (CMS),

TU Wien, Wiedner Hauptstraße 8-10, A-1040 Wien, Austria

(Dated: November 27, 2016)

* emanuela.bianchi@tuwien.ac.at

† gerhard.kahl@tuwien.ac.at

I. STABILITY CHECKS

We have applied two complementary simulation techniques (described in the Methods section of the manuscript) to study (i) the formation of the layered structure and its self-healing capacities (see subsection I A), and (ii) the coexistence and the thermal stability of competing phases (see subsection I B).

A. On the formation of the layers

In order to test the self-assembly capacities of the system, we have launched Molecular Dynamics simulations from completely random particle configurations (with respect to boh the positions and the orientations of IPCs). These simulations demonstrated that for $T \lesssim 0.12$ IPCs are always able to self-assemble into the layered structure. It should be noted that the density of the emerging ordered structure has always values close to the ideal density (i.e., $\rho^* \simeq 0.75$), while the remaining volume of the system is filled with a very low density gas (i.e., $\rho^* \ll 0.01$), reflecting the extremely broad gas-solid coexistence region (see the phase diagram displayed in the left panel of Figure 3 of the manuscript). A typical configuration of the system at such coexistence conditions is shown in Figure 1 of this document.

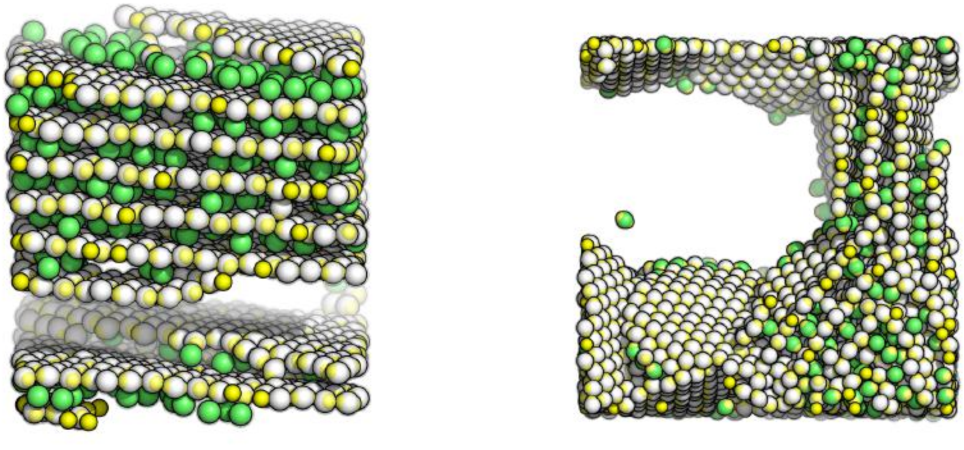

FIG. 1. Snapshots of self-assembled structures taken from MD simulations (left: $\rho^* = 0.70$ and $T^* \approx 0.060$; right: $\rho^* = 0.40$ and $T^* \approx 0.060$); at this state points the crystal-liquid phase (with different layer orientations) is in coexistence with the disordered gas phase (isolated particles).

Analyzing numerous movies based on particle trajectories that were computed in our

MD simulations, we could identify the strategy through which our IPCs self-assemble into the ordered layered structure. Starting from a relatively small planar cluster of particles (mutually arranged in the grain-like structure depicted in the top panel of Figure 2 of the manuscript), an extended layer grows rather rapidly and is soon able to span the entire simulation volume. At this stage the eminent role of the inter-layer particles in the structure formation process comes into play: they adsorb on the surface of the newly formed layer, creating strong bonds with the layer particles in the optimal equator-patch arrangement. With their orientations being perpendicular to the plane these inter-layer IPCs provide, in turn, anchoring points for still mobile particles, which start to build – again via the same equator-patch bonding mechanism – the hexagonally closed-packed network of the next layer (which is, by construction, parallel to the previous one). At this level, the process is repeated until the entire layered structure is formed.

In some cases, during the early stage of the simulation, two (sometimes more) layers can simultaneously form in different regions of the simulation box; apart from some exceptional occurrence, they are not be parallel to each other. In these cases, one of the layers turns out to be faster in creating other parallel layers. The emerging ordered structure grows at the cost of the slowest layers: the particles of the non-aligned layers are dissolved from these planar arrangements and are incorporated step-by-step to build up the dominant structure (see the corresponding movie: in the the movie particles are colored according to their final identity: if, at the end of the movie, they belong to the layers then they are colored in grey throughout the movie, otherwise they are colored in green). This self-healing process is very frequently observed at densities closer to the ideal value (i.e. for densities down to $\simeq 0.70$). At smaller densities (i.e., typically $\rho^* \lesssim 0.6$) it often occurs that the number of mobile particles is not high enough to foster the self-healing process. In such cases, stacks of layers emerge that are characterized by different orientations (see Figure 1 of this document).

It is worth noting that also intentionally destroying an existing layered structure during a simulation run triggers the self-healing mechanism: particles that are liberated during the destruction process either (i) take over the role of inter-layer particles and quickly arrange as anchoring points on existing layers or (ii) they contribute as layer particles to the lateral grows of the layers.

It should be also emphasized that above $T^* \simeq 0.08$ defects can emerge.

B. On the stability of the layers

Direct coexistence Monte Carlo simulations were used to (i) identificate the exact temperature ranges in which the respective phases are found to be stable and (ii) confirm the equilibrium value of the stoichiometric ratio between inter-layer and layer particles (i.e., a value of 2/7).

To tackle the first issue, half of the simulation box was filled with the hybrid crystal-liquid structure at its ideal density, i.e. $\rho^* = 0.75$, while the other half of the box contained the liquid phase at the same density of the layered phase. The system was then let evolve during a simulation at a fixed temperature. Figure 2 shows typical snapshots taken at temperatures below and above the melting transition. Below the transition temperature ($T^* \leq 0.13$) the entire simulation box is eventually filled with the layered structure (upper panels), while above the transition temperature ($T^* \geq 0.14$) the crystal melts and the entire system is found to be in the fluid phase. Thus we can conclude that the melting temperature is in the range $0.13 \leq T^* \leq 0.14$.

To address the question about the value of the stoichiometric ratio between inter-layer and layer particles, the initial two-phase configuration was built such that the average density of the whole system was either smaller or larger than the ideal density of the ordered phase: we achieved that by reducing the number of inter-layer particles in the initial layered architecture; at the same time the ordered structure and the liquid always had the same density at the beginning of the simulation. For both smaller and larger average densities, the stoichiometric ratio between inter-layer and layer particles was observed to equilibrate during the simulation runs and reach the value 2/7: when $\rho^* < 0.75$, particles are sucked from the liquid phase to the inter-layer cavities, thus reducing the density of the fluid phase till the ordered phase reaches its equilibrium density; when $\rho^* > 0.75$ the excess inter-layer particles in the ordered phase are expelled into the liquid phase.

II. PARTICLE DYNAMICS

The dynamics of the particles was quantified via their (partial) mean squared displacements and their orientational autocorrelation functions, defined and discussed in the following.

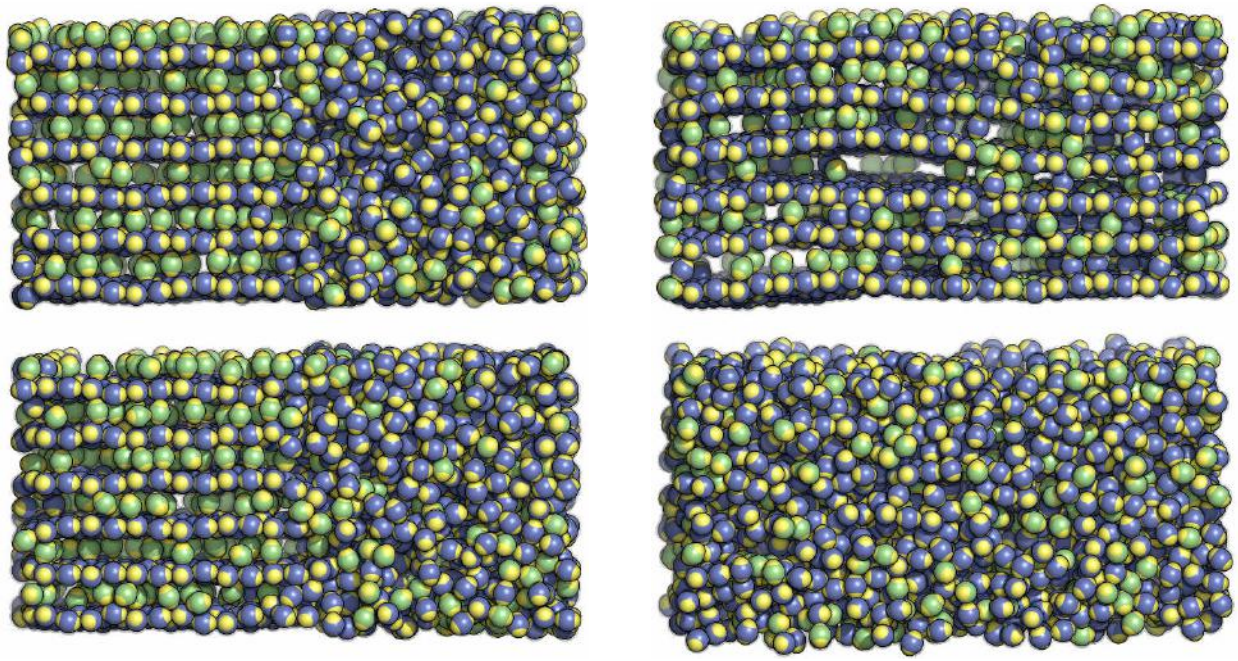

FIG. 2. Snapshots from Monte Carlo direct coexistence simulations to bracket the transition temperature between the (semi-)ordered layered phase and the liquid at $\rho^* = 0.75$. Upper panels correspond to a temperature below the melting transition ($T^* = 0.12$), while lower panels show snapshots at a temperature above the transition ($T^* = 0.14$). Left panels are initial configurations, right panels are equilibrated final configurations.

A. The mean squared displacements (MSDs)

Within the temperature range where the MSD was calculated ($0.015 \leq T^* \leq 0.115$), the parallel layers are essentially stiff, planar constructs; this fact allows us to embed an (x, y)-plane in a sheet formed by the particles, with the z-axis obviously pointing into a direction perpendicular to the plane.

We are thus able to split the MSD, $\Delta \mathbf{r}^2(t)$, into two contributions that specify its lateral and perpendicular components:

$$\Delta \mathbf{r}^2(t) = \Delta x^2(t) + \Delta y^2(t) + \Delta z^2(t) = \Delta (xy)^2(t) + \Delta z^2(t). \quad (1)$$

From the data shown in the different panels of Figure 3 we can learn the following about the *layer particles*: with their $\Delta (xy)^2$ and Δz^2-values being throughout the smallest ones among all MSDs displayed, these particles form essentially rigid, immobile layers, with the

particles vibrating only around their respective equilibrium orientations; this holds essentially for all temperatures up to $T^* \simeq 0.072$. Only for the highest temperature investigated (i.e., for $T^* \simeq 0.115$, i.e., close to the melting temperature of the system) these partial MSDs show slightly increased values, indicating that the layer particles gain some mobility.

In contrast, from the different MSDs of the *inter-layer particles* we can learn that they are essentially frozen in their positions only for $T^* \lesssim 0.045$. Above this threshold value we observe that Δz^2 is still characterized by rather small values (i.e., throughout $\lesssim 10^{-2}$ squared diameters), indicating that no transport of inter-layer particles perpendicular to the planes takes place; while $\Delta(xy)^2$ quickly grows with increasing the temperature, providing evidence for a growing particle transport in the lateral direction (as discussed in the manuscript).

It should be noted that in the simulation runs for the two highest temperature values (i.e., for $T^* = 0.095$ and 0.115) an increasing number of particles were able to switch from their original position within a layer to some inter-layer position and vice-versa. Since we used as the criterium to discriminate between layer and inter-layer particles a sizable value of their corresponding, individual MSDs, we are unable to exclude these (few) "switching IPCs" from the sampling process and they were considered as inter-layer IPCs.

B. Orientational autocorrelation functions (OACFs) – full time range

While Figure 3 of the manuscript shows the OACFs over a limited time range (improving thereby the visibility of the oscillations of these functions at short times), such a representation precludes a quantitative discussion of the long-time decay of these correlation functions. The corresponding data (recorded over the entire simulation duration, i.e., for $t^* \leq 1000$) are shown in Figure 4 of this document. We observe that the decay of the long-time tail of the OACFs of the inter-layer particles is ten times slower than that of the layer particles.

At the lowest temperature, i.e. $T^* = 0.015$ (panel H), the factor of ten between the decay times of the two species can be readily confirmed. At $T^* = 0.072$ (panel E), we observe that the OACF of the layer particles shows exactly the same oscillations as the corresponding function calculated for the lowest temperature; in contrast, the inter-layer OACF decays faster (even though still moderate on an absolute scale) and flattens at a value close to 0.90. Finally, at the highest temperature, i.e. $T^* = 0.115$ (panel B), the OACFs of both types of particles show a rapid initial decay and then saturate to values close to 0.50. We note that

the above mentioned problems in strictly discriminating layer and inter-layer particles has also some influence on the correlation functions.

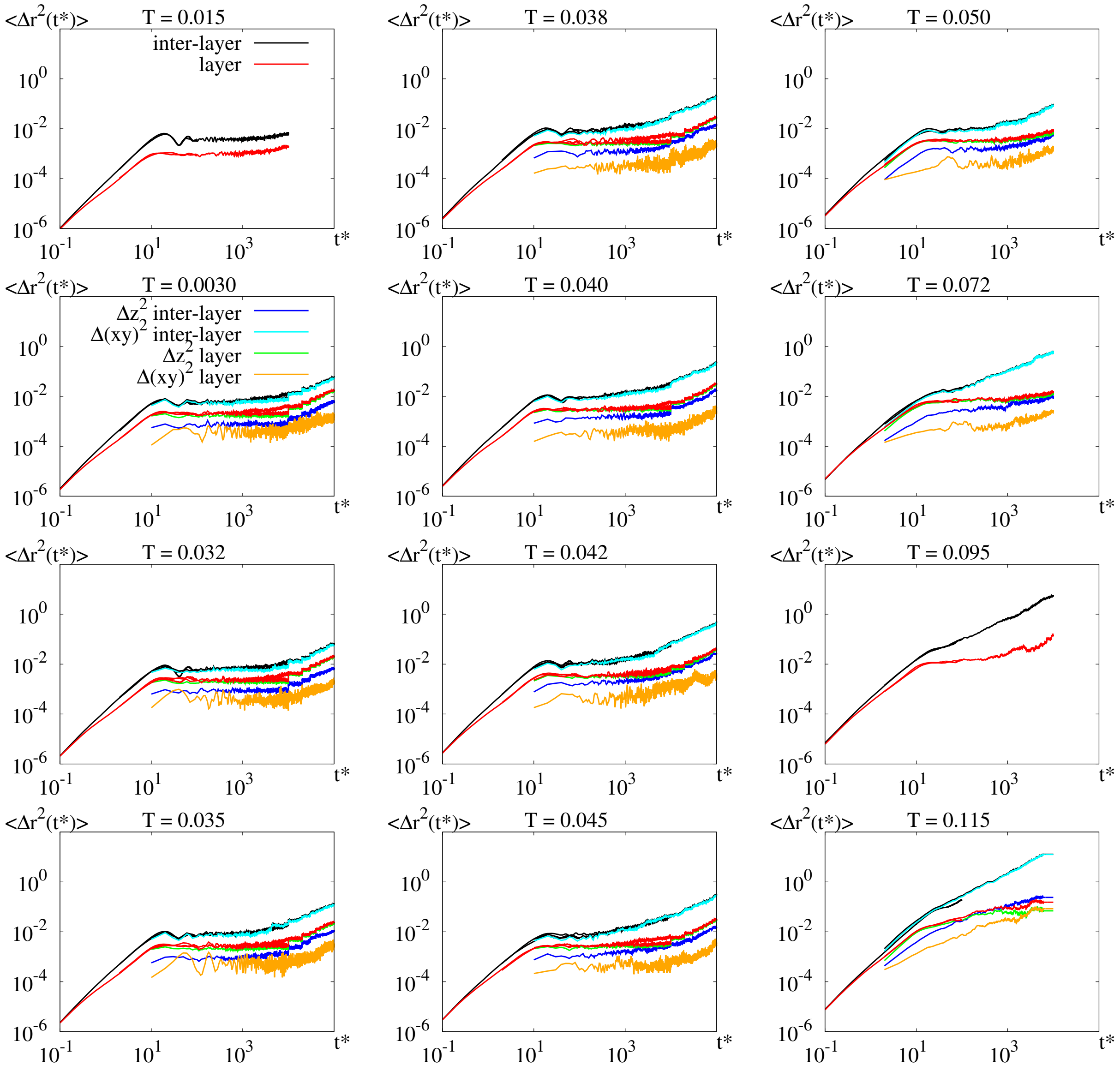

FIG. 3. Various types of mean squared displacement (MSD) at selected temperatures (as labeled) as functions of time: (i) full MSD (i.e., $\Delta \mathbf{r}^2(t)$) for the inter-layer (blue lines) and for the layer (red lines) particles, respectively; (ii) lateral and perpendicular contributions to the MSD (i.e., $\Delta(xy)^2$ and Δz^2, as defined in Equation (1)), for the inter-layer and the layer particles, respectively (color code as specified in one of the panels). Most of the different types of MSDs have been sampled up to $t^* = 10^5$; however, in some cases they were only available up to $t^* = 10^4$.

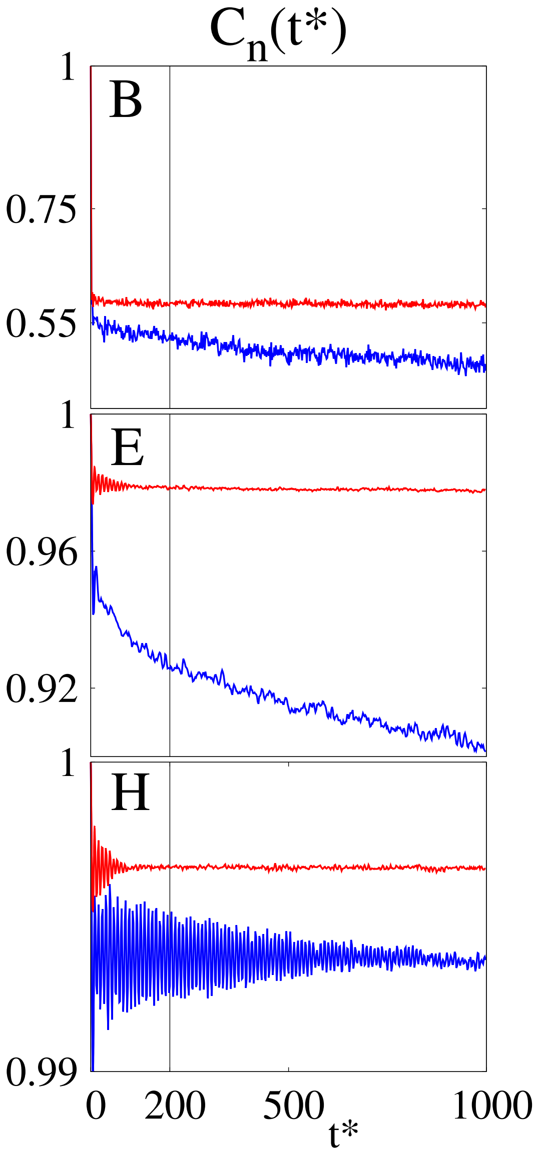

FIG. 4. OACFs of layer (red) and inter-layer particles at $T^* = 0.115$ (panel B), $T^* = 0.072$ (panel E), and $T^* = 0.015$ (panel H), displayed over the entire time range that was investigated in our MD simulations, i.e., for $t^* \leq 1000$. The vertical thin lines at $t^* = 200$ indicate the time range over which the OACFs are displayed in Figure 3 of the manuscript.
